# Case report of an ST-elevation Myocardial Infarction-like presentation of an immune checkpoint (PD-1/PD-L1) inhibitor-associated myocarditis

**DOI:** 10.1093/ehjcr/ytae429

**Published:** 2024-08-16

**Authors:** Astrid Declercq, Stefan Verstraete, Lieve Vanwalleghem, Sander Trenson

**Affiliations:** Cardiology Resident, Department of Cardiology, Ghent University Hospital, Corneel Heymanslaan 10, 9000 Ghent, Belgium; Department of Cardiology, General Hospital Zeno, Kalvekeetdijk 260, 8300 Knokke, Belgium; Department of Anatomical Pathology, General Hospital Sint-Jan, Ruddershove 10, 8000 Bruges, Belgium; Department of Cardiology, General Hospital Sint-Jan, Ruddershove 10, 8000 Bruges, Belgium

**Keywords:** Immune checkpoint inhibitor, Myocarditis, Myocardial infarction, Case report

## Abstract

**Background:**

ICI-associated myocarditis is a rare but severe and potentially life-threatening complication that typically manifests shortly after treatment initiation. It may present in many different ways, ranging from fulminant to non-fulminant, even including clinical and electrocardiographic findings mimicking ST-elevation Myocardial Infarction (STEMI).

**Case summary:**

A 72-year-old woman with a history of non-small cell lung carcinoma presented at the emergency department with symptoms of general asthenia and chest pain, following recent ICI-therapy initiation. Electrocardiogram showed ST elevation in the lateral leads and led to prompt admission for urgent invasive coronary angiography, which ruled out significant coronary artery disease. Urgent cardiac magnetic resonance had to be aborted due to claustrophobia. Endomyocardial biopsy—performed the day after urgent hospital admission and before starting high-dose corticosteroids—confirmed acute ICI-associated myocarditis. On the sixth day of hospitalization, the patient developed transient complete heart block and non-sustained ventricular tachycardia, necessitating temporary transjugular pacemaker insertion. Cellcept (mycophenolate mofetil) was associated due to rising troponin levels. Following a three-week hospital stay, the patient was discharged with a regimen of gradually tapering steroids and continued Cellcept therapy. Two months post-discharge, the patient was readmitted due to severe pneumonia, ultimately resulting in the patient’s demise.

**Discussion:**

We present the case of a fulminant ICI-associated myocarditis. The case illustrates the diagnostic workup and treatment strategies of an (in the end) fatal adverse event from the use of immune checkpoint inhibitors.

**Learning points**

- ICI-associated myocarditis is a rare yet potentially fatal complication that can manifest in diverse ways, ranging from fulminant to non-fulminant. Its prevalence may be underestimated due to underreporting of milder cases.
- It is recognized that the clinical and electrocardiogram findings in a patient with ICI-associated myocarditis may mimic those of an ST-Elevation Myocardial Infarction at initial presentation.
- Our case report illustrates current ESC guidelines concerning the diagnostic approach and therapeutic strategies in ICI-associated myocarditis.

## Introduction

Immune checkpoint inhibitors (ICIs), such as PD-1/PD-L1 inhibitors, are increasingly utilized in the management of various cancers, including those affecting the lung, skin, and kidney.

ICIs function as immunotherapeutic agents by targeting proteins that inhibit the immune system’s ability to combat cancer cells. Although the pathophysiology is not clearly defined, ICIs may also trigger an overactivation of T-cells against non-cancerous tissues, leading to immune-related adverse events. Myocarditis is a rare (0.3–1.1%) but severe and potentially life-threatening complication that typically manifests shortly after treatment initiation.^1^ It may present in many different ways, ranging from fulminant to non-fulminant, and may exhibit clinical and electrocardiogram (ECG) findings similar to ST-Elevation Myocardial Infarction (STEMI).

## Summary figure

**Figure:**
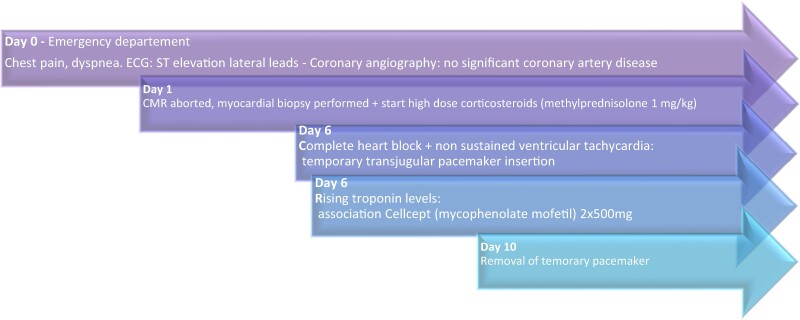


## Case presentation

**Figure 1:**
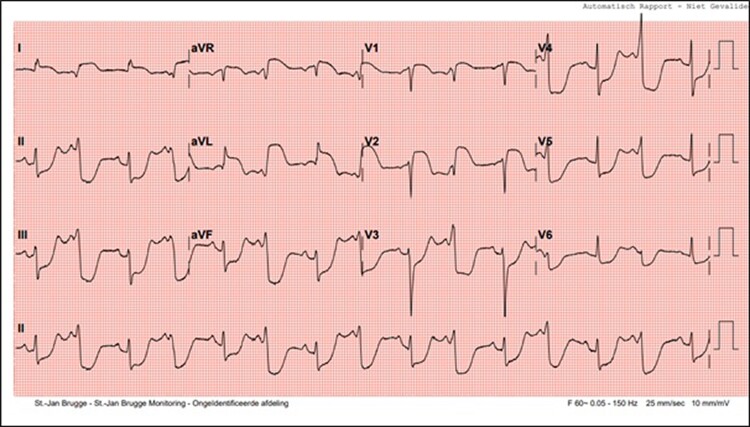
Twelve-lead electrocardiogram at presentation.

A 72-year-old female patient presented to the emergency department on 1 November 2021, with symptoms of general malaise, nausea, diarrhoea, chest pain, and bilateral shoulder pain. Upon initial assessment, her vital signs included a heart rate of 90 beats/min, blood pressure of 159/61 mmHg, respiratory rate of 20/min, and oxygen saturation of 94% on room air. Physical examination revealed a regular heart rate without murmurs and no signs of congestion such as oedema, jugular venous distension, or lung crackles. The electrocardiogram obtained at presentation (*Figure 1*) displayed ST-segment elevations in the lateral and precordial V1–V2 leads, accompanied by reciprocal ST-segment depressions in the inferior leads and precordial V3–V6 leads.

She had a medical history of type 2 diabetes, arterial hypertension, obesity, chronic obstructive pulmonary disease and stage IIIa non-small cell lung carcinoma, for which she had received concomitant chemo(carboplatinum/taxol)-radiotherapy, resulting in complete remission as confirmed by PET-CT imaging. Subsequently, on 7 October 2021, she initiated consolidation therapy with an immune checkpoint inhibitor: Imfinzi (durvalumab)—a PD-1/PD-L1 inhibitor.

**Figure 2:**
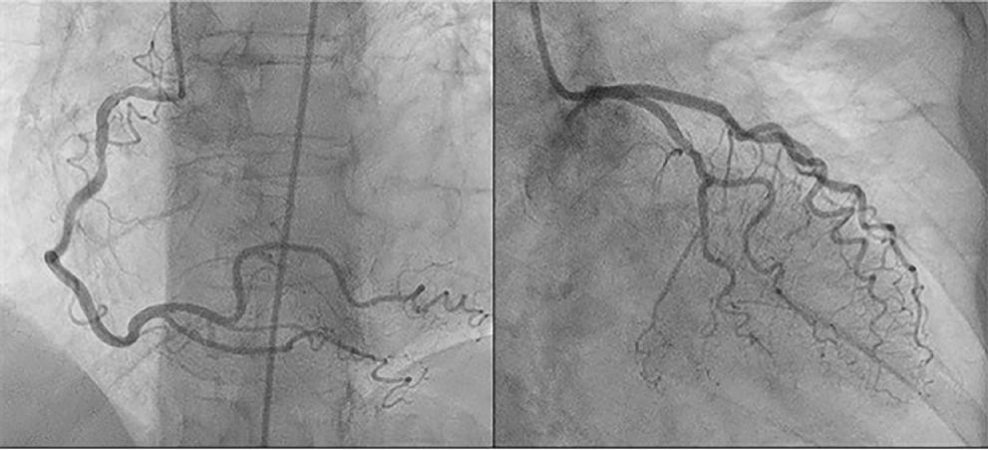
Results of the coronary angiography, showing the right coronary artery (RCA) on the left side and the left coronary artery (LCA) and circumflex artery (Cx) on the right side.

**Figure 3:**
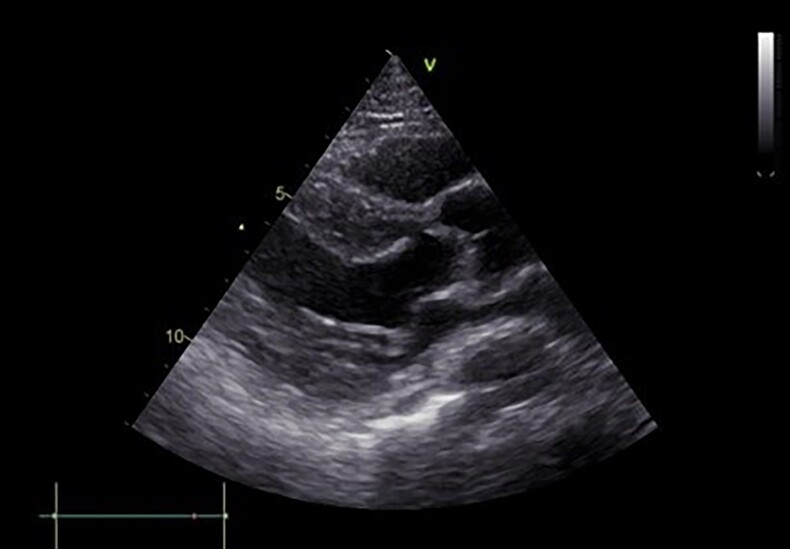
Parasternal long axis (PLAX) view of the transthoracic echocardiography, showing concentric left ventricular hypertrophy.

She was promptly referred for urgent invasive left coronary angiography, demonstrating the absence of significant coronary artery lesions (*Figure 2*). The laboratory analyses revealed elevated cardiac enzymes [Troponin I = 14 000 ng/L and NTproBNP (N-terminal probrain natriuretic peptide) = 6000 pg/mL]. C-reactive protein was measured at 13 mg/L. Echocardiography showed concentric hypertrophy of the left ventricle (interventricular septum thickness 16 mm), with preserved ejection fraction and no significant valvular disease (*Figure 3*) (see Supplementary material online, *Video S1*).

**Figure 4:**
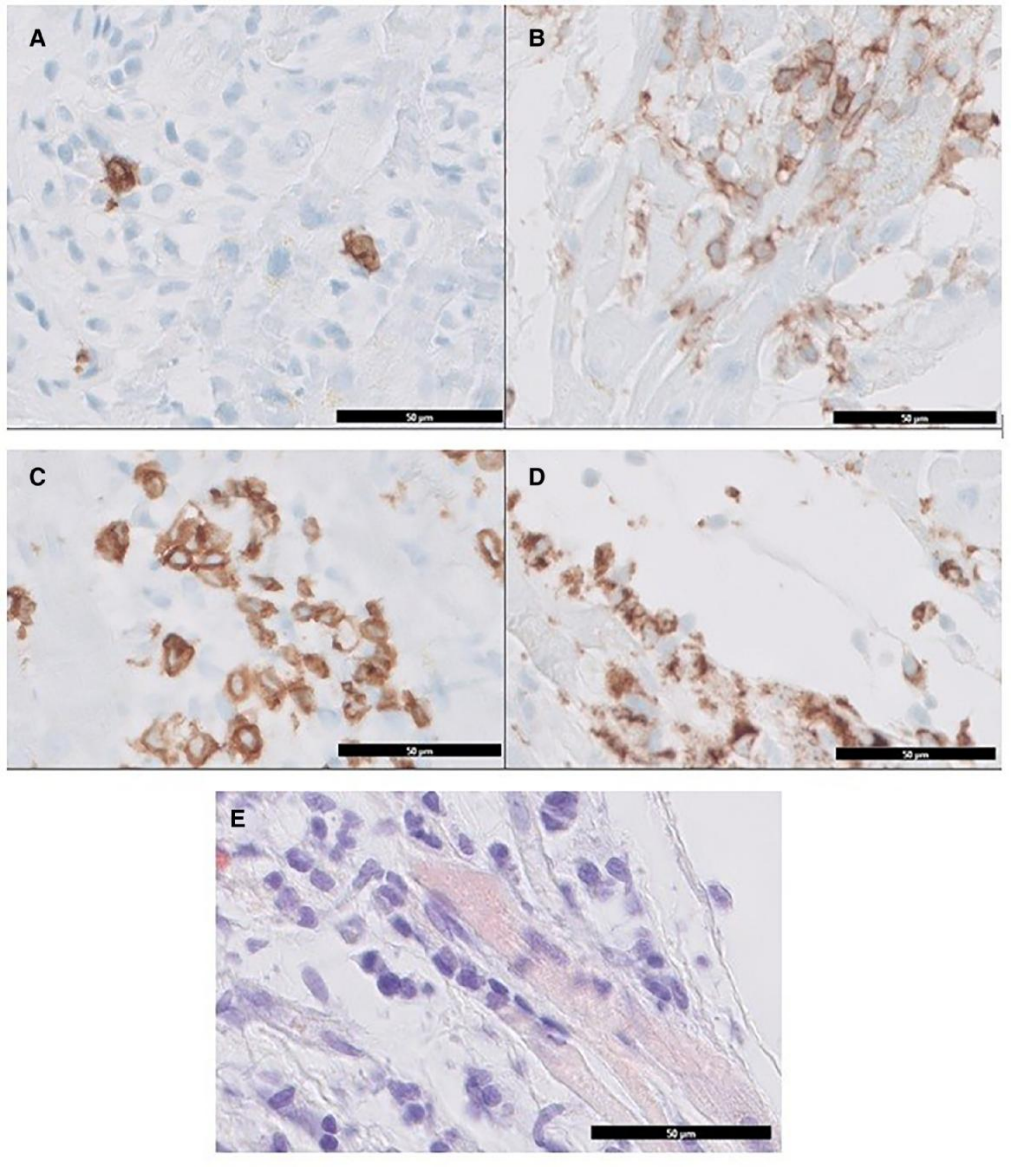
Results from the anatomopathological examination of the endomyocardial biopsy. (*A*) CD20 expression, marking B-lymfocytes; (*B*) CD45 (protein tyrosine phosphatase) expression, marking leucocytes; (*C*) CD3 expression, marking T-lymfocytes; (*D*) CD68 expression, marking monocytes and macrophages. (*E*) Congo red staining, for enhanced visualization of eosinophilic granulocytes. Scale bar: 50 μm.

Cardiac MRI was aborted two times due to claustrophobia. Prior to the initiation of high-dose steroids (methylprednisolone 1 mg/kg), an endomyocardial biopsy (EMB) was performed. The biopsy results confirmed the presence of acute myocarditis, characterized by a predominance of CD4^+^ T-lymphocytes, a hallmark feature of ICI-associated myocarditis (*Figure 4*). Immune checkpoint inhibitor therapy was discontinued.

**Figure 5:**
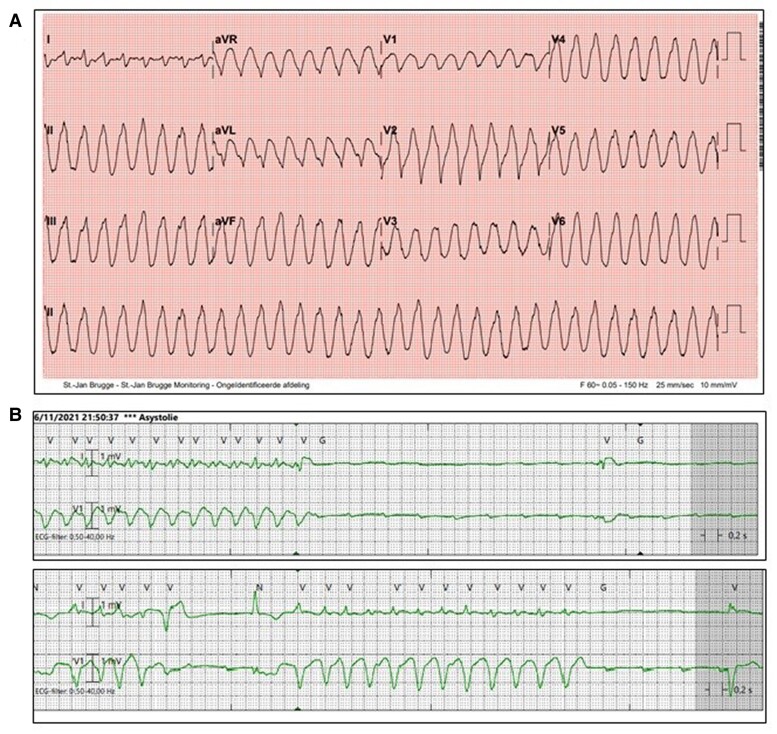
(*A*) Twelve-lead electrocardiogram on day 6, showing an episode of non-sustained ventricular tachycardia (NSVT). (*B*) Cardiac telemetry showing NSVT with underlying complete heart block.

On day 6, the patient developed multiple arrhythmias, including transient complete heart block, followed by multiple episodes of non-sustained ventricular tachycardia (*Figure 5*). A transjugular temporary pacemaker was inserted, with stabilization of the arrhythmias. Concurrently, there was again a significant rise in troponin levels—while echocardiographic observations remained consistent (indicating left ventricular hypertrophy with preserved ejection fraction) and corticosteroid doses were still unaltered (methylprednisolone 1 mg/kg)—prompting the association of Cellcept (mycophenolate mofetil) at a dosage of 2 × 500 mg. The decision not to escalate the dosage of methylprednisolone (e.g. 1000 mg/daily for 3 consecutive days) was made after multidisciplinary team deliberation (considering potential benefits vs. elevated infection risk).

The temporary pacemaker was successfully withdrawn after a span of 4 days (on day 10 of hospitalization), and troponin levels declined. During further in hospital monitoring, no more arrhythmias appeared. The left ventricular wall thickness stabilized and reduced to 14 mm prior to discharge (see Supplementary material online, *Videos S2* and *S3*). Heart failure therapy consisted of ramipril (ACE-inhibitor). Beta-blocker therapy was withheld due to recent complete heart block. SGLT2-inhibitor was not yet initiated (and was at that moment—for patients with heart failure with preserved ejection fraction, HFpEF—only available in a medical need program).

Our patient experienced steroid-induced side-effects including myopathy, asthenia, and dysregulation of glycaemia. Nevertheless, discharge was deemed feasible following a three-week hospitalization period.

Cellcept administration persisted alongside a slow steroid dosage tapering (methylprednisolone 48 mg upon discharge, tapering to 40 mg after 4 weeks, and so forth). Ambulatory follow-up with echocardiography and troponin assay would decide further tapering speed.

The dosage of Cellcept was intended to remain unchanged until the complete normalization of troponin levels. Pneumocystis prophylaxis was given 3 times/week (sulfamethoxazol 800 mg +trimethoprim 160 mg).

Two months following discharge, the patient was readmitted due to severe pneumonia, leading to respiratory failure necessitating ventilatory support. Difficult weaning with repetitive need of urgent re-intubation resulted in a protracted ICU stay, ultimately requiring tracheostomy placement. Nevertheless, after a two-month hospitalization period, progressive respiratory insufficiency recurred. Considering the unfavourable prognosis, exacerbated by overall debilitation and in alignment with the patient’s wishes, further attempts at intubation were abstained from. Palliative care was initiated, and the patient died shortly thereafter.

## Discussion

Myocarditis can present with symptoms and electrocardiographic (ECG) changes similar to those seen in ST-Eelevation Myocardial Infarction (STEMI), leading to diagnostic challenges and potential misinterpretation. The occurrence of myocarditis mimicking STEMI is rare, with an estimated clinical diagnostic incidence of 0.17 per 1000 man-years.^2^ It occurs particularly in younger individuals or those with a recent history of viral illness or immune-related therapy.

Our patient was diagnosed with a fulminant ICI-associated myocarditis, typically occurring early after initiation (most frequently during the first 12 weeks) of ICI therapy.^3^ The diagnosis of ICI-associated myocarditis is initially based on the presence of symptoms, a new increase in troponin, and new ECG abnormalities.^3^ Transthoracic echocardiography (TTE) and cardiac magnetic resonance imaging (CMR) are recommended in all patients to confirm diagnosis, while EMB should be considered in cases where the diagnosis is suspected but not confirmed non-invasively.^3,4^

Given the expanding application of ICI across diverse malignancies, heightened vigilance from both oncologists and cardiologists is warranted, regarding the increasing incidence of both fulminant and non-fulminant ICI-associated myocarditis. The true incidence of ICI-associated myocarditis may be underestimated due to the underreporting of milder cases. According to current ESC cardio-oncology guidelines, cardiovascular assessment, including ECG, troponin and NTproBNP assays, as well as TTE, are recommended prior to initiation of ICI therapy.^3,5^ Follow-up with ECG and troponin assay should be considered (class IIa recommendation).^3,5^

Current guidelines recommend discontinuation of ICI therapy and treatment with high-dose corticosteroids. In haemodynamically unstable patients (ventricular arrhythmias or complete heart block) with high clinical suspicion of myocarditis, treatment with high-dose methylprednisolone should always promptly be initiated (without waiting for CMR and/or EMB results).^3^

If insufficient effect of steroid treatment, other immunosuppressive agents are suggested. There remains a lack of evidence to guide appropriate immunosuppressive therapy in ICI-induced myocarditis, as current guidelines are based on expert consensus. No randomized controlled trials have been performed.^3^ In case of heart failure or cardiogenic shock, common supportive treatment strategies apply (i.v. diuretics, inotropes, mechanical support devices). Arrhythmias may occur and can be treated with a temporary pacemaker, cardioversion, or pharmaceutical interventions.^3^

In summary, this case illustrates the rare presentation of ICI-associated myocarditis mimicking ST-Elevation Myocardial Infarction. Further diagnostic approach and treatment options are discussed—yet should be evaluated on a case-by-case scenario—due to the broad range in clinical presentation of ICI-associated myocarditis. Our patient was successfully stabilized with corticosteroid and mycophenolate mofetil therapy, together with temporary transjugular pacing. Yet, 2 months after discharge, she suffered fatal severe pneumonia, probably due to immunocompromised status. This indicates the high mortality of fulminant ICI-associated myocarditis, even after surviving the acute phase.

## Supplementary Material

ytae429_Supplementary_Data

## Data Availability

The data underlying this article will be shared on reasonable request to the corresponding author.

## Lead author biography

Dr Declercq Astrid is a last year trainee in Cardiology at Ghent University Hospital. She has a special interest in Interventional Cardiology.
